# Biejiajian Pill Promotes the Infiltration of CD8^+^ T Cells in Hepatocellular Carcinoma by Regulating the Expression of CCL5

**DOI:** 10.3389/fphar.2021.771046

**Published:** 2021-11-26

**Authors:** Xuemei Yang, Jialing Sun, Bin Wen, Yu Wang, Mingjia Zhang, Weicong Chen, Wenting Zhao, Chunyu He, Xiaodan Zhong, Yang Liu, Tong Li, Haitao Sun, Songqi He

**Affiliations:** ^1^ Nanfang Hospital, Southern Medical University, Guangzhou, China; ^2^ School of Traditional Chinese Medicine, Southern Medical University, Guangzhou, China; ^3^ Department of Traditional Chinese Medicine, Hospital of PLA, Guangzhou, China; ^4^ Department of Hepatobiliary Surgery, Nanfang Hospital, Southern Medical University, Guangzhou, China

**Keywords:** hepatocellular carcinoma, traditinal Chinese medicine, CD8^+^T cell, CCL5, Biejiajian Pill

## Abstract

Tumor-infiltrating CD8^+^T lymphocytes are mostly associated with a favorable prognosis in numerous cancers, including hepatocellular carcinoma (HCC). Biejiajian Pill (BJJP) is a common type of traditional Chinese medicine that is widely used in the treatment of HCC in China. Previous studies showed that BJJP suppressed the growth of HCC cells both *in vivo* and *in vitro*, by exerting direct cytotoxic effects on tumor cells. The present study demonstrated that in addition to direct cytotoxicity, BJJP inhibits the growth of tumor cells by promoting the infiltration of CD8^+^T cells into the tumor in H_22_-bearing mice. Mechanistically, chemokine ligand 5 (CCL5) was identified as one of the most highly expressed chemokines by tumor cells *in vivo* after treatment with BJJP. Additionally, CCL5 was knocked down in H_22_ cells and the results showed that knockdown of the gene significantly impaired the infiltration of CD8^+^T cells *in vivo*. Furthermore, the effects of BJJP on human HCC cell lines were assessed *in vitro*. Similarly, cells treated with BJJP had higher expression of CCL5 mRNA, which was consistent with increased levels of CCL5 protein in human tumor cells. These findings provide new insights into the anticancer effects of BJJP, which regulated the expression of CCL5 and the infiltration of CD8^+^T cells. The results, therefore, suggest that BJJP has great potential application in clinical practice.

## Introduction

Hepatocellular carcinoma (HCC) is the most common type of liver cancer and is associated with approximately one million deaths globally each year (Llovet et al., 2021). Nonetheless, most HCC patients have benefited from the current treatment options, including surgery, radiofrequency ablation, liver transplantation, immunotherapy and neoadjuvant chemoradiotherapy (Kumari et al., 2018). However, all these approaches are associated with several limitations. Therefore, new therapeutic options for HCC are urgently needed. Notably, combination therapy using traditional Chinese medicine (TCM) is an important treatment strategy for HCC (Ling et al., 2018; Tang et al., 2020; Zhao et al., 2020; Gou et al., 2021). This approach has numerous advantages as it inhibits tumor growth, relieves complications from surgery, prevents adverse reactions from drugs, enhances the quality of life and improves the overall 5-year survival rate of patients with advanced HCC (Qi et al., 2015; Yang et al., 2017; Liao et al., 2020).

More specifically, Biejiajian Pill (BJJP), initially recorded in “Synopsis of Prescriptions of the Golden Chamber,” is a well-known and classical Chinese medicine formula. The formula was originally used to treat hepatosplenomegaly caused by malaria, with multiple benefits, including promoting a healthy qi, removing toxins, dissolving knots and promoting blood circulation to remove blood stasis (Xu and Liu, 2020). It was also shown that BJJP inhibits the growth and invasiveness of HCC cell lines by targeting different cellular signaling pathways, including Wnt/β-catenin (Sun et al., 2014), RhoA/ROCK (An et al., 2018) and Akt/GSK-3β/Snail (Sun et al., 2021). Its molecular mechanism was also associated with the inhibition of Epithelial–mesenchymal transition (EMT), which promoted tumor cells migration and affected the recurrence and prognosis of HCC (Li et al., 2021). In addition, previous studies demonstrated that BJJP exerts antitumor effects in HCC patients at different stages of the disease (Ping et al., 2008; Yao, 2009; Zheng et al., 2017).

It is also well-known that tumor-infiltrating CD8^+^ T lymphocytes confer a favorable prognosis in various types of cancers, including bladder (Sharma et al., 2007), colon (Ling et al., 2014), colorectal (Galon et al., 2006), esophageal (Gao et al., 2020), pancreatic (Carstens et al., 2017), breast (Mahmoud et al., 2011) and liver (Kondratiev et al., 2004) cancers. However, whether BJJP inhibits the growth of HCC by regulating tumor immunity has scarcely been explored. Herein, we investigated the effects of BJJP on the infiltration of CD8^+^ T cells and the related mechanism in HCC cells and the H_22_ subcutaneous graft tumor model. The findings revealed that BJJP inhibits the growth of tumor cells in an immune-dependent manner. The results also showed that BJJP promotes the infiltration of CD8^+^ T cells into HCC tumors in H_22_-bearing mice or in an *in vitro* co-culture system. Mechanistically, BJJP was shown to regulate the expression of chemokine ligand 5 (CCL5) both *in vivo* and *in vitro*. Furthermore, knockdown of CCL5 in H_22_ cells significantly impaired the infiltration of CD8^+^ T cells *in vivo*. The study also assessed whether BJJP can regulate the expression of CCL5 in human HCC cell lines, using SMMC-7721 and HepG2 cells. The results revealed that treatment with BJJP increased CCL5 levels in tumor cells. These findings provide new information on the anticancer effects of BJJP, which was shown to regulate the expression of CCL5. BJJP, therefore, has great potential application in clinical practice in the treatment of HCC.

## Materials and Methods

### Cells and Culture

The mouse HCC-derived H_22_ cell line, as well as the human HCC-derived HepG2 and SMMC-7721 cell lines, were purchased from the Type Culture Collection of the Chinese Academy of Sciences (Shanghai, China). The H_22_ cells were maintained in the RPMI-1640 medium, while the HepG2 and SMMC-7721 cells were maintained in the DMEM medium. The media were supplemented with 10% fetal bovine serum and the cells were kept at 37°C in a humidified atmosphere containing 5% CO_2_. PBMC was obtained from NanFang hospital research center, which was test negative for *mycoplasma*, HIV, syphilis and hepatitis virus.

### Mouse Model and BJJP Treatment

Female BALB/c mice or BALB/c-nu/nu mice aged 5–6 weeks were provided by the Guangdong medical laboratory animal center (Guangzhou, China). The mice were fed in pathogen-free facilities with standard food and tap water. To establish a mouse model, H_22_ cells (5 × 10^5^) were harvested in 150 µl of phosphate-buffered saline (PBS) and transplanted subcutaneously into the right flank of the mice. After 24 h, the mice were given either normal saline or BJJP (3 g/kg, Sinopharm Zhonglian Pharmaceutical Co., Ltd. Wuhan, China). In addition, tumor size was monitored using a caliper and the mice were weighed daily. Mice were sacrificed on day 21. Four mice in “shRNA CCL5+Ctrl” and “shRNA CCL5+BJJP” group were sacrificed before terminal of experiment because of poor survival condition and body weight loss than 30%. Thereafter, tumors from each mouse were harvested, weighed, imaged then fixed in paraformaldehyde for further analysis. All the animal experiments were approved by the Institutional Animal Care and Use Committee (IACUC) of Southern Medical University.

### Preparation of Serum Containing BJJP

Serum-containing BJJP was prepared according to a previously published protocol (Sun et al., 2021). The rats were randomly divided into the BJJP-low dose (L) and BJJP-high-dose (H) (0.55 and 2.2 g/kg, respectively, dissolved in sterilized normal saline) and negative control (NC) groups. Serum containing BJJP was then collected and stored at −80°C.

### TUNEL Staining for the Detection of Tumor Apoptosis

To detect tumor apoptosis, TUNEL staining was performed using a TUNEL assay kit (C1088, Beyotime), according to the manufacturer’s protocol. Briefly, formalin-fixed paraffin-embedded tissues were sectioned, dewaxed, and treated with the Proteinase K solution for antigen retrieval. Thereafter, the sections were immersed in the membrane-breaking fluid (0.1% Triton X-100 diluted in PBS) for 20 min. After reaching an equilibrium at 37°C for 10 min, the sections were maintained in the TUNEL reaction mixture at 37°C for 2 h, in the dark. Afterward, they were washed thrice using PBS and then counterstained with DAPI for another 10 min. Finally, the sections were treated with an anti-fade mounting medium, then visualized using a fluorescence microscope to obtain photomicrographs.

### Preparation of Single Cells From Subcutaneous Tumors

To analyze the infiltration of CD8^+^ T lymphocytes into subcutaneous tumors, collagenase IV was used to digest single cells, as previously described (Zhang et al., 2018). Briefly, the tumors were first cut and then digested with collagenase IV for 1 h, at 37°C (17104–019, Gibco). Thereafter, the dissociated cells were strained through a 70-μm nylon mesh (352350, BD). Then, cells were centrifuged for 8 min at 350 g at 4 °C, resuspended in PBS.

### Migration of CD8^+^ T Cells *in Vitro* Using Transwell Assay

The effect of BJJP on the migration of CD8^+^ T cells was assessed by FCM. The mouse or human HCC cells were plated in 24-well plates and then treated with 10% designated concentrations (L, H) of BJJP. Thereafter, 1 × 10^6^ spleen cells or PBMC were plated in the upper chamber with 5.0 μm-sized pores (3421, Corning) at 37°C for 48 h. Afterward, all cells in the bottom chamber were stained with anti-CD8a antibody. Migrating CD8^+^ T cells were then detected by FCM.

### Knockdown of CCL5 Using shRNA

Lentiviruses containing mouse shCCL5 (5′-CTC​CAA​TCT​TGC​AGT​CGT​GTT-3′) and an shRNA control (Genechem, Shanghai, China) were transfected into H_22_ cells. Cells with stable knockdown of CCL5 were then exposed to 5 μg/ml of puromycin (Sigma, United States). Thereafter, the efficacy of shCCL5 knockdown was verified by Western blot analysis.

### Immunofluorescence Staining

All cells treated with BJJP or tumor samples were first fixed, blocked and incubated overnight with anti-CCL5 (1:200; Bioss, bs-54125R) or anti-CD8 (1:100; Bioss, bs-0648R) antibodies, at 4°C, followed by incubation with Alexa Fluor™568 conjugated secondary antibodies (1:500; Thermo Fisher; A11011) at room temperature for 60 min, in the dark. Finally, the distribution of CCL5 or CD8 fluorescence was visualized using a fluorescence microscope (Nikon, Tokyo, Japan) or Dragonfly highspeed confocal microscopy (ANDOR, Oxford Instruments).

### Fluorescence-Activated Cell Sorting

For flow cytometry analysis, cultured cells were harvested and stained for 15 min at room temperature using CD8-PE-Cy7 (BD, 561097). For the isolation of CD8^+^ T cells form subcutaneous tumors, single cells were digested by collagenase IV and filtered through 70-µm and 40-µm filters. Then, cells were centrifuged for 8 min at 350 g at 4 °C, resuspended in PBS, and stained for 20 min at room temperature using CD8-PE-Cy7 (BD, 561,097, 1:250). DAPI was used to exclude dead cells. Next, for T-cell effector function analysis, the Harvested cells were stimulated with 1 μM ionomycin (Sigma, I3909) and 50 ng/ml phorbol 12-myristate 13-acetate (PMA, Sigma, P1585) for 4 h. Intracellular staining and flow cytometry were used to measure TNF-α-PE (Biolegend, 506305), IFN-γ-APC (Biolegend, 505809) or GranzymeB-PE (Biolegend, 372207) productions by CD8^+^ T cells. Samples were detected by Flow cytometry (Beckman Coulter Cytoflex) and the data was analyzed using the CytExpert software (Beckman).

### Enzyme-Linked Immunosorbent Assay

Mouse serum CCL5 and supernatant CCL5 derived from the BJJP-treated cancer cell lines were analyzed using respective immunoassay kits, the mouse CCL5 ELISA kit (MM-0881M2, MEIMIAN) and the human CCL5 ELISA kit (MM-14376H2, MEIMIAN). Thereafter, an ELISA kit was used to detect the levels of CCL5, according to the manufacturer’s protocol.

### Real-Time PCR Analysis

Total RNA was extracted using the Trizol reagent (ER501-01, TransGen, China), following the manufacturer’s protocol. Thereafter, the PrimeScript™ RT reagent kit with a gDNA Eraser (RR047a, Takara, Japan) was used for first-strand cDNA synthesis. In addition, quantitative real-time PCR (qPCR) analysis was performed on a Light Cycler^®^96 System (Roche Applied Science, Germany) using the SYBR^®^ Premix Ex Taq™II (Tli RNaseH Plus) kit (RR820a, Takara, Japan). Changes in mRNA expression were then calculated as fold changes, using the 2^−△△^CT method. The mouse or human-specific gene primers are detailed in Supplementary Table S1.

### Western Blotting

Western blot analysis was conducted using a standard protocol. Briefly, protein extracts from cultured cells and tumor tissues were lysed in RIPA lysis buffer containing protease and phosphatase inhibitors (all purchased from Beyotime, Shanghai, China). Thereafter, the protein lysates were separated by 12% sodium dodecyl sulfate-polyacrylamide gel electrophoresis (SDS-PAGE), and then transfer-embedded onto polyvinylidene difluoride (Millipore, United States) membranes. This was followed by incubation with 5% bovine serum albumin. Afterward, the membranes were probed with specific primary antibodies against CCL5 (1:1,000; Bioss bsm-54125R) and GAPDH (1:1,000; Servicebio GB11002), followed by incubation with horseradish peroxidase-conjugated secondary antibodies. Finally, the banks were detected by enhanced chemiluminescence (ECL; CST).

### Statistical Analysis

All data were analyzed and mapped using GraphPad Prism 8. In addition, comparisons between groups were made using the unpaired Student’s *t*-test or one-way ANOVA, with Tukey’s multiple comparisons test. A *p*-value < 0.05 was considered statistically significant.

## Results

### BJJP Promotes Antitumor Immunity in Hepatocellular Carcinoma

**FIGURE 1:**
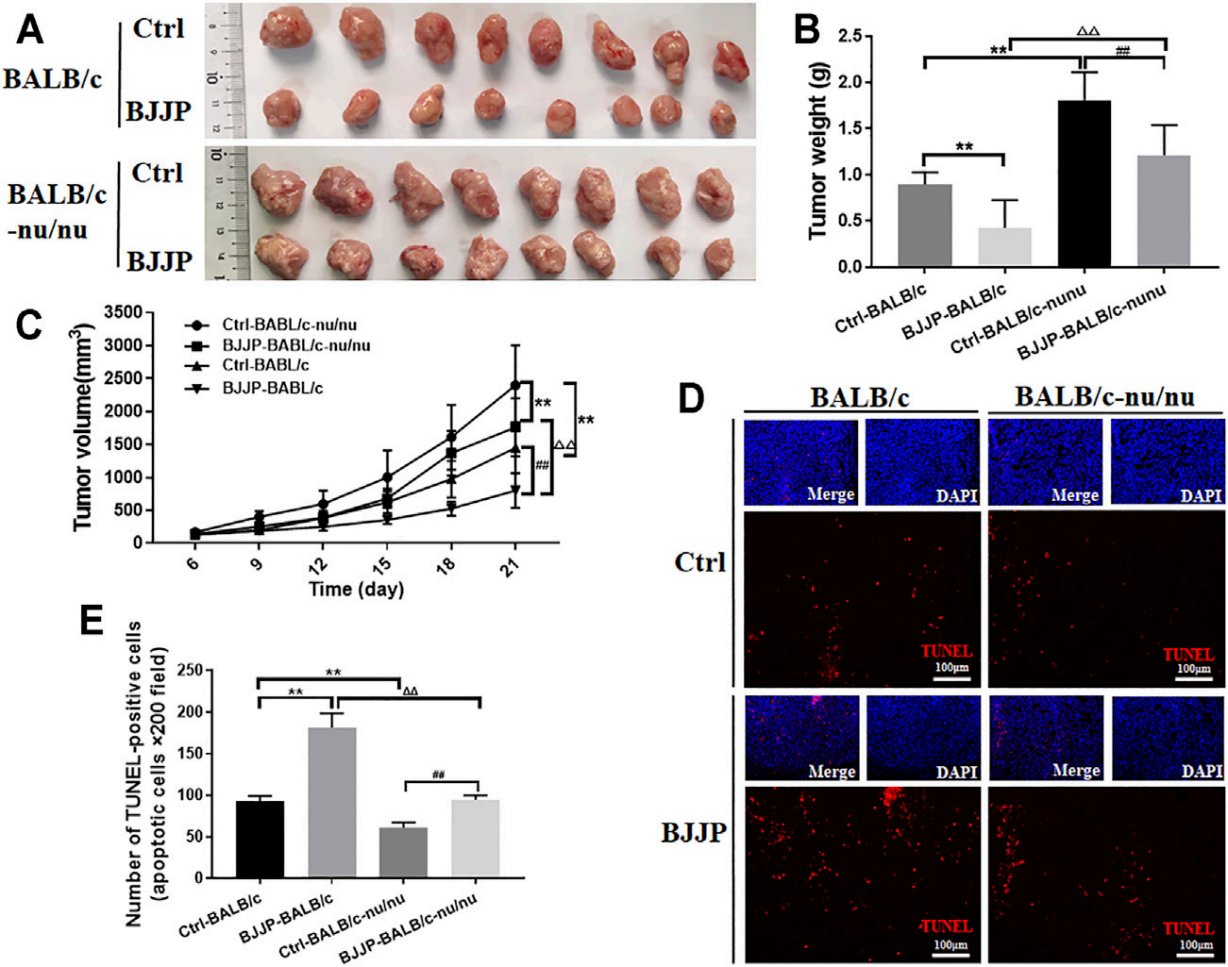
BJJP promotes antitumor immunity in HCC. **(A)** BALB/c-nu/nu or BALB/c mice were injected with H_22_ cells after which they received a daily intragastric gavage of BJJP or saline for 21 consecutive days. Subcutaneous tumor tissues were then obtained from the mice and photographed (*n* = 8). **(B,C)** Quantification of tumor weights and volumes. **(D,E)** TUNEL staining of the tumor tissues (scale bar, 100 μm, 200×). The mean ± SD of three independent experiments is shown for each sample. **p < 0.01 vs BALB/c controls; ##*p* < 0.01 vs BALB/c-nu/nu mice controls; ΔΔ*p*<0.01 vs. BALB/c mice BJJP.

To explore the effect of BJJP on antitumor immunity, antitumor effects of BJJP in immunodeficient BALB/c-nu/nu and immunocompetent BALB/c mice were compared using the H_22_ tumor cell model. The results showed that mice treated with BJJP had reduced cell growth compared to the controls, suggesting that BJJP exerted antitumor effects in both immunodeficient (BALB/c-nu/nu) and immunocompetent (BALB/c) mice (Figures 1A–C). However, tumor weights and volumes in the immunocompetent BALB/c mice were lower than those in the immunodeficient BALB/c-nu/nu mice, after treatment with BJJP. Notably, the weight of tumors in the BJJP-treated BALB/c-nu/nu mice was approximately 2 times that of BJJP-treated BALB/c mice (Figures 1B,C). Next, TUNEL staining was used to detect apoptosis of cells in tumor tissues. As expected, the BJJP-treated immunocompetent BALB/c mice had the highest rate of apoptosis (Figures 1D,E). Moreover, BJJP treatment showed less cytotoxicity on liver tissues and immortalized liver LO_2_ cells (Supplemental Figures S4A, B). These results therefore strongly suggest that BJJP can promote antitumor immunity in hepatocellular carcinoma.

### BJJP Promotes Infiltration of CD8^+^ T Cells *in Vivo*

**FIGURE 2:**
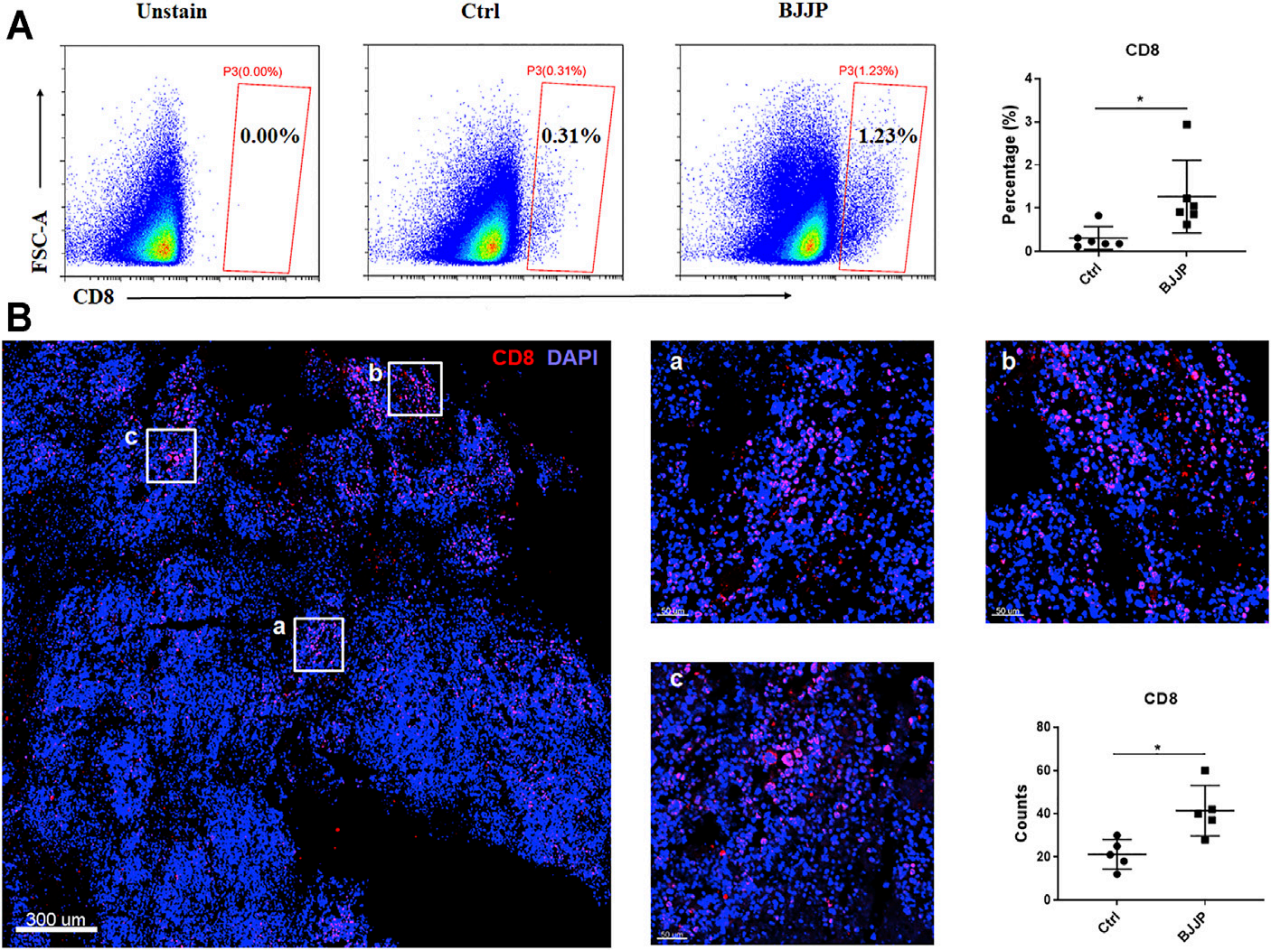
BJJP promotes the infiltration of CD8+ T cells *in vivo*. **(A)** Data showing the percentage of CD8^+^ T cells after tumor digestion. **(B)** Immunostaining of CD8^+^ T cells (red) in BJJP treated group. The total CD8^+^ T cell count from five randomly selected fields per mouse was analyzed. The mean ± SD of three independent experiments is shown for each sample. *p < 0.05 vs. controls.

CD8^+^ T cells are thought to play a key role in tumor immunity, and infiltration of CD8^+^ T lymphocytes into solid tumors is correlated with a favorable prognosis in various types of cancers. Therefore, to investigate the role of BJJP in the infiltration of CD8^+^ T cells in HCC, tumor tissues from immunocompetent BALB/c mice were examined by immunofluorescence or fluorescence-activated cell sorting (FACS). The findings showed that the proportion of CD8^+^ T cells was higher in the BJJP-treated BALB/c group after tumor digestion, suggesting more CD8^+^ T cells infiltration after treatment with BJJP (Figure 2A). Results from immunofluorescence staining also confirmed that a larger number of CD8^+^ T cells were infiltrated into the tumor, compared to the control group (Figure 2B and Supplemental Figure S1A). In addition, BJJP treatment also promoted the effector function of CD8^+^T cells by significant increasing the expression of TNF-α and IFN-γ in tumor-infiltrating CD8^+^ T cells (Supplemental Figure S3B, C).

### BJJP Enhances the Expression of CCL5 *in Vivo*

Since chemokines and chemokine receptors are necessary for the recruitment of CD8^+^ T cells into tumors, the mRNA expression levels of various chemokines secreted by H_22_ tumor cells were assessed, including *CCL5, CCL9, CCL17, CXCL9, CXCL10* and *CXCL12*, which were identified to regulate the migration of CD8^+^ T cells in previous studies (Berghuis et al., 2011; Zhang et al., 2020).

**FIGURE 3:**
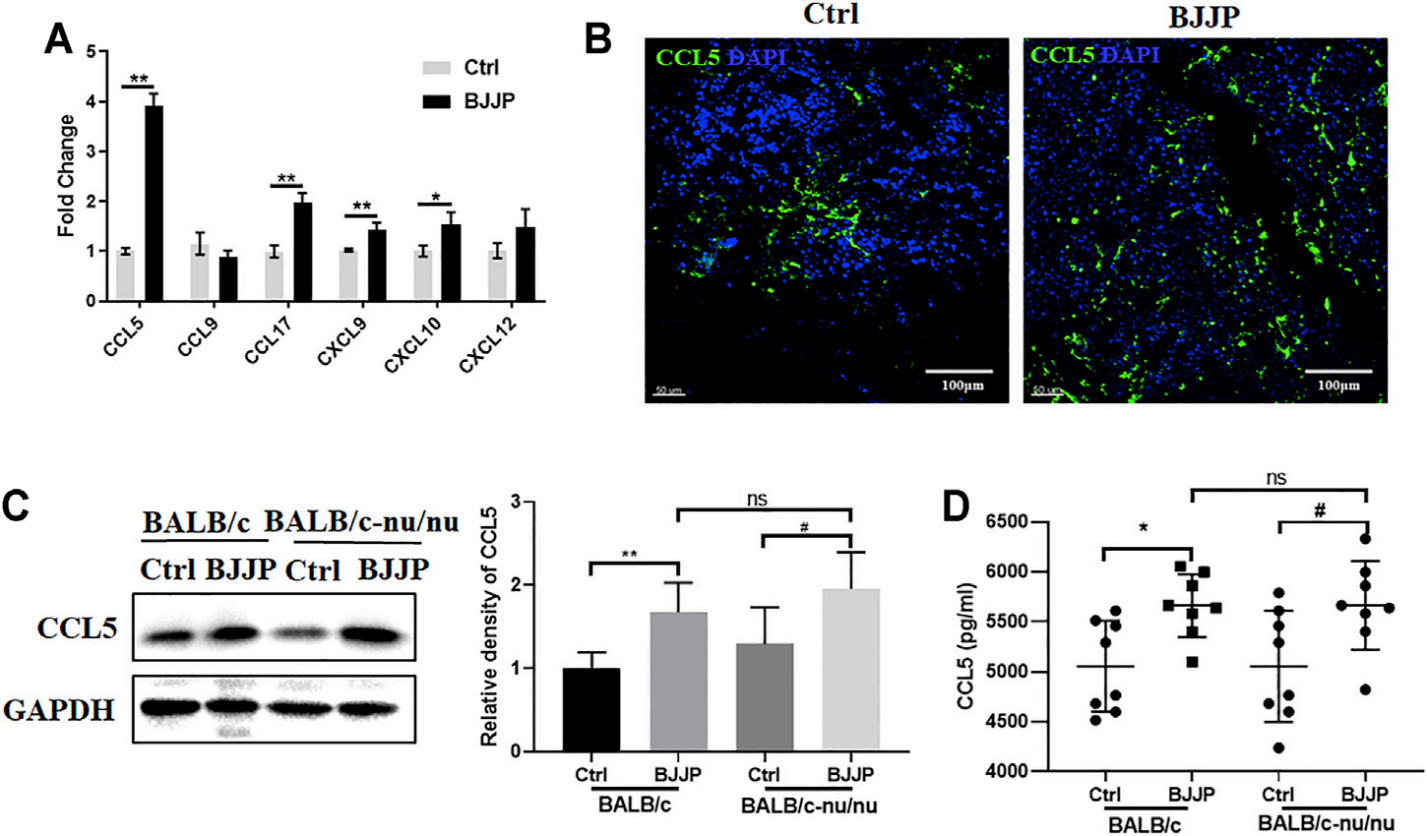
BJJP enhances the expression of CCL5 *in vivo*. **(A)** Relative mRNA levels of *CCL5, CCL9, CCL17, CXCL9, CXCL10* and *CXCL12*. The expression of CCL5 in tumor tissues was examined using **(B)** immunofluorescence and **(C)** Western blotting. **(D)** Levels of CCL5 in mice serum were assessed by ELISA. The mean ± SD of three independent experiments is shown for each sample. *p < 0.05, **p < 0.01 vs. controls; ^#^ *p* < 0.05 vs. CCL5-Ctrls.

GFP^+^ H_22_ cells were harvested from tumor-bearing mice by FACS sorting, for mRNA detection on day 21 after the injection of tumor cells (Supplemental Figure S1B). Among the assessed chemokines, *CCL5* exhibited the most remarkable change in expression between the control and BJJP-treated groups (Figure 3A). Immunofluorescence staining and Western blotting also confirmed that there was a significant increase in the expression of CCL5 in the subcutaneous tumors of mice in the BJJP group (Figures 3B,C). When co-stained CCL5 with CD8^+^ T cells, we found fields with higher CCL5 expression showed more CD8^+^ T cells infiltration (Supplemental Figure S2A). Additionally, higher levels of serum CCL5 were observed in the BJJP-treated group (Figure 3D).

### BJJP Enhances the Expression of CCL5 *in Vitro*

**FIGURE 4:**
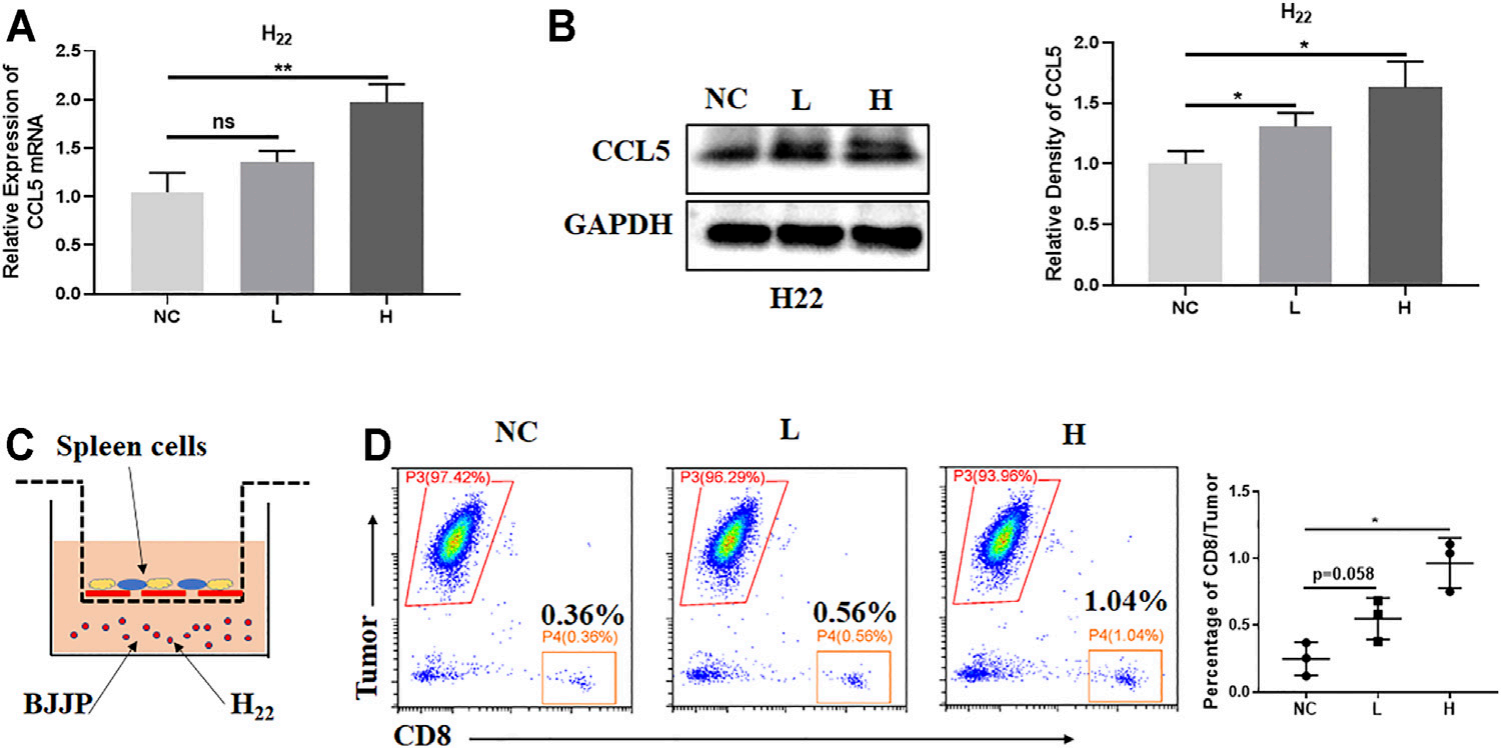
BJJP enhances the expression of CCL5 *in vitro*. H_22_ cells were exposed to designated concentrations of BJJP (L, H) for 24 h. **(A,B)** Increased mRNA and protein levels of CCL5, respectively, after treatment with BJJP for 24 h. **(C)** A schematic diagram of the spleen cell migration model. **(D)** Migrating CD8^+^ T cells were detected by flow cytometry. The data represents the mean ± SD of technical triplicates. *p < 0.05, **p < 0.01 vs. controls.

To uncover the role of BJJP on H_22_ cells *in vitro*, serum-containing BJJP was prepared from Wistar rats, and NC, low dose (BJJP-low) and high dose (BJJP-high) groups were assessed. Interestingly, the results showed that BJJP enhanced the expression of CCL5 in a dose-dependent manner (Figures 4A,B). Next, *in vitro* experiments were used to evaluate the migration of murine CD8^+^ T cells towards tumor cells. Therefore, 1 × 10^6^ spleen cells from BABL/c mice were co-cultured with GFP^+^H_22_ cells in the upper Transwell. After co-culture for 48 h, the cells were harvested from the lower wells then the percentage of CD8^+^ T cells was calculated using FCM. The results revealed a significant increase in the number of migrated CD8^+^ T cells in the BJJP-treatment groups, and that migration occurred in a dose-dependent manner (Figures 4C,D).

### Inhibiting the Expression of CCL5 Reverses the Effects of BJJP

**FIGURE 5:**
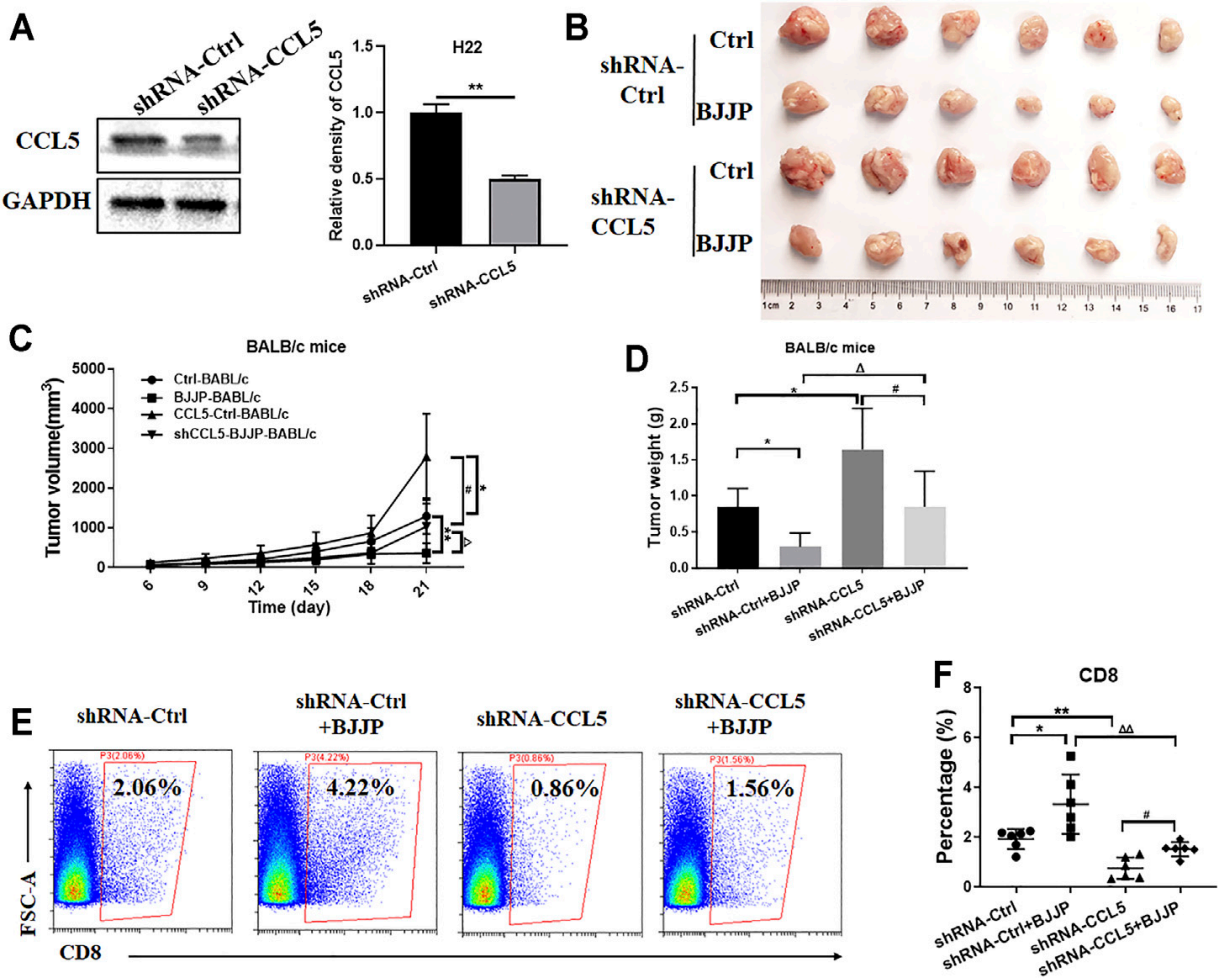
Inhibiting the expression of CCL5 reverses the effects of BJJP. **(A)** Western blotting was used to detect the relative expression of CCL5 in H_22_ cells transfected with shRNA-CCL5 or shRNA-Ctrl. The data is presented as the mean ± SD from three independent experiments. H_22_ cells in which CCL5 was knocked down were injected subcutaneously in BALB/c mice. The subcutaneous tumors obtained from the mice were then **(B)** photographed, **(C)** measured and **(D)** weighed. **(E,F)** The percentage of CD8^+^ T cells after tumor digestion was obtained using flow cytometry. Data are presented as the mean ± SD (*n* = 6). **(A)** **p < 0.01 vs. controls. **(C,D)** *p < 0.05, **p < 0.01 vs controls; ^##^ *p* < 0.01 vs. CCL5-Ctrls; ^Δ^ *p* < 0.05, ^ΔΔ^ *p* < 0.01 vs. BALB/c mice BJJP.

To confirm whether BJJP regulates the infiltration of CD8^+^ T cells by enhancing the expression of CCL5, CCL5 was knocked down in H_22_ cells using RNA interference. The results confirmed that shRNA-CCL5 successfully reduced the expression of CCL5 in H_22_ cells (Figure 5A). Next, tumor growth was evaluated in immunocompetent BALB/c mice. Compared to the BJJP-treated normal H_22_ cells, the effect of BJJP was significantly impaired in the BJJP-treated H_22_-CCL5 knockdown category, as depicted by the decrease in the size and weight of tumors (Figures 5B–D). In addition, there was a significant decrease in the infiltration of CD8^+^ T cells in the BJJP-treated H_22_-CCL5 knockdown group although the number was still higher than that in the BJJP-untreated category (Figures 5E,F).

### BJJP Regulates CCL5 in Human HCC Cells

**FIGURE 6:**
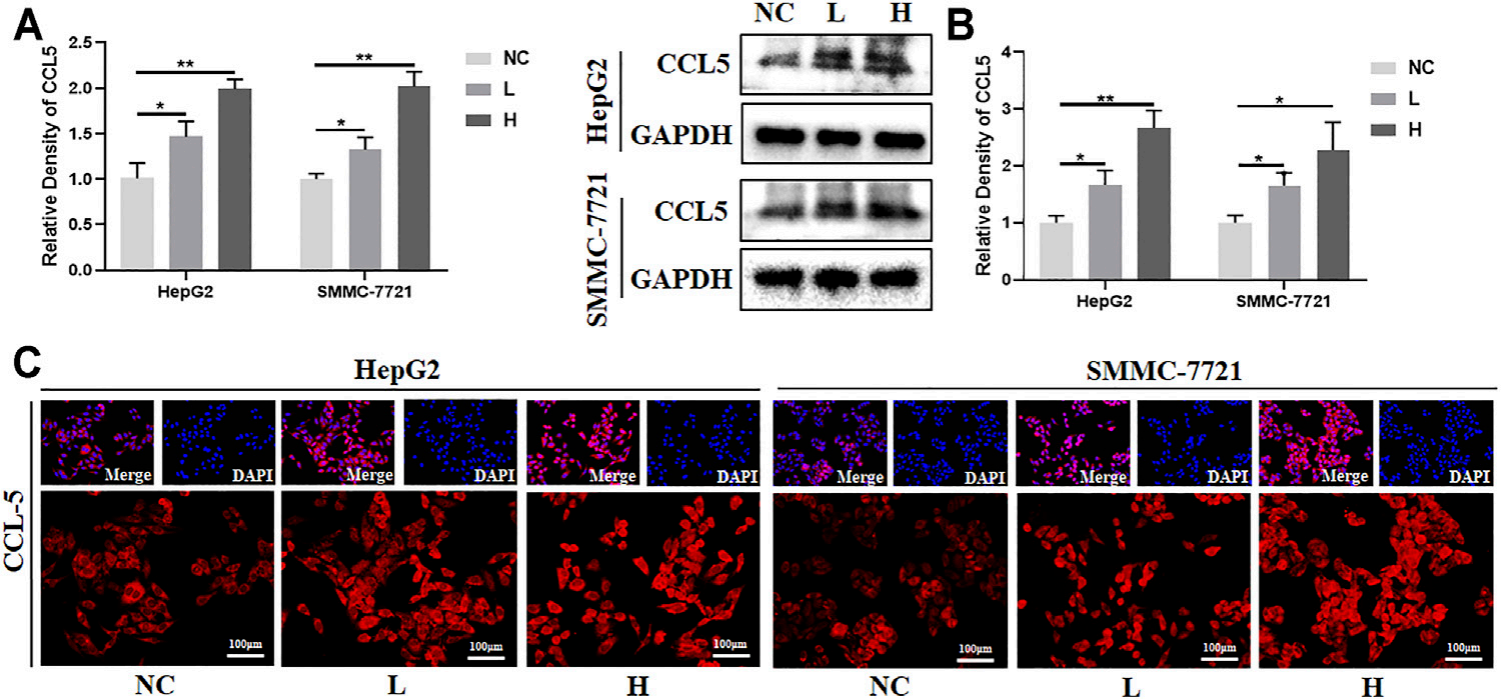
BJJP regulates CCL5 in human HCC cells. The HepG2 and SMMC-7721 human hepatocellular carcinoma cells were exposed to designated concentrations of BJJP (L, H) for 24 h. **(A,B)** Increased mRNA and protein levels of CCL5, respectively, after treatment with BJJP for 24 h. **(C)** The distribution of CCL5 fluorescence in HepG2 and SMMC-7721 cells was examined using a fluorescence microscope (scale bar, 100 μm, 200×). The mean ± SD of three independent experiments is shown for each sample. *p < 0.05, **p < 0.01 vs. controls.

To validate the findings in the mouse model, the effects of BJJP on human SMCC-7721 and HepG2 cells were analyzed. As expected, there was a 2- and 3-fold increase in the mRNA and protein expression levels of CCL5, respectively, in the BJJP-treated group (Figures 6A,B). The results from immunostaining also showed that there was an increase in the expression of CCL5 in the BJJP-treated HCC cells (Figure 6C). Next, the expression of CCL5 in the culture supernatant was assessed using ELISA. The protein level of CCL5 in the supernatant was nearly undetectable. To evaluate the migration of human CD8^+^ T cells towards tumor cells, 1x10^6^ human PBMC cells were co-cultured with SMCC-7721 or HepG2 cells in the upper transwell. There was a significant increase for the infiltration of CD8^+^ T cells with BJJP treatment (Supplemental Figure S2B). Overall, these findings suggest that BJJP could inhibit tumor growth clinically may also through promoting the infiltration of CD8^+^ T lymphocytes.

## Discussion

BJJP is a canonical formula from “The Synopsis of Golden Chamber” that is made up of 23 ingredients and is widely used in TCM for the treatment of HCC. A previous study by our research group showed that the main components of BJJP were wogonin, zerumbone, ursolic acid, resveratrol and 6-gingerol (Sun et al., 2021), all of which have been reported to regulate tumor immunity. Notably, wogonin was shown to have immune-modulatory effects as it regulated the function of effector T cells (Fan et al., 2020). Moreover, zerumbone can regulate immune responses and inflammation through the MAPK and NF-κB pathways (Haque et al., 2017). It was also shown that ursolic acid can enhance the production of IL-2 and IFN-gamma (Jang et al., 2009). Resveratrol was reported to improve the efficacy of radiotherapy by enhancing anti-tumor immunity (Kim et al., 2020). Furthermore, the administration of 6-gingerol suppressed tumor growth *in vivo* by enhancing the inflation of tumor-infiltrating lymphocytes (Ju et al., 2012). Previous study found that BJJP exerted a tight inhibitory influence on the progression of HCC by the inhibition of EMT, which plays important role in hepatocellular carcinoma (Li et al., 2021; Sun et al., 2021). It was reported that EMT signature is inversely associated with T-cell infiltration, which promote tumor cell to escape from immune system defense (Chae et al., 2018; Aghajani et al., 2020). However, whether BJJP exerts its antitumor effects by regulating tumor immunity is yet to be elucidated. Therefore, the present study sought to investigate the effects of BJJP on the infiltration of CD8^+^ T cells in HCC and the related mechanism. The findings revealed that BJJP promotes the infiltration of CD8^+^ T cells into HCC tumors in H_22-_bearing mice and in an *in vitro* co-culture system. As it is well known that PD-L1 was characterized as an immune regulatory molecule, which inactivated CD8^+^T cells for immune escape. The present study showed that BJJP had no effect on the expression of PD-L1 in tumor cells (Supplemental Figure S3A). Then, we measured the effector function of CD8^+^ T cells. As expected, BJJP could promote the effector function of CD8^+^T cells by regulating the production of cytokines TNF-α and IFN-γ. Mechanistically, there was a significant increase in the expression of CCL5 in the BJJP-treated group. Additionally, knockdown of CCL5 in H_22_ cells significantly impaired the infiltration of CD8^+^ T cells *in vivo*. The results also showed that BJJP regulated the expression of CCL5 in human HCC cell lines. These findings, therefore, provide new information on the anticancer effects of BJJP, which regulates the expression of CCL5 and the infiltration of CD8^+^ T cells.

Tumor-infiltrating CD8^+^ T cells are associated with disease progression in cancers, and cytotoxicity of CD8^+^ T cells has spurred great interest in cancer immunotherapy (Fu et al., 2007; Yang et al., 2016). In addition, an increase in the number of CD8^+^ T cells or enhanced anti-tumor effector functions in the tumor microenvironment may predict a good prognosis. Nonetheless, the molecular mechanisms underlying the infiltration of CD8^+^ T cells in solid tumors are complicated. One of the classical mechanisms involves chemokines, which regulate the infiltration of immune cells in tumors (Dangaj et al., 2019). Notably, several chemokines, including CCL5, CCL9, CCL17, CXCL9, CXCL10 and CXCL12, have been found to regulate the migration of CD8^+^ T cells into HCC or other solid tumors (Berghuis et al., 2011; Zhang et al., 2020).

CCL5, also known as RANTES, was reported to be produced by cancer cells or nonmalignant stromal cells in the tumor microenvironment (Singh et al., 2018). Previous studies showed that CCL5 was exerted by binding to CCR5 and could promote the growth, migration and invasiveness of pancreatic cancer or HCC cells *in vitro* (Mohs et al., 2017; Singh et al., 2018; Xue et al., 2021). Additionally, it was also reported that reduction of CCL5 expression caused tumor-infiltrating lymphocyte (TIL) desertification and forced CCL5 expression prevent TIL loss *in vivo* (Dangaj et al., 2019). But, the function of CCL5 on HCC *in vivo* has never been mentioned. When we generated a CCL5 knockdown H_22_ cell line and implant to tumor bearing mice, the tumor weight in shRNA CCL5-Ctrl mice was higher than shRNA Ctrl-Ctrl, which suggested CCL5 may have an anti-tumor effect (Figure 5D). Furthermore, we confirmed that BJJP treatment induced expression of CCL5 *in vivo*, which facilitate CD8^+^T cell infiltration (Figures 2A,B). Next, we demonstrated that inhibiting the expression of CCL5 reversed the effects of BJJP (Figures 5E,F). It is therefore possible that BJJP exerted a tight anticancer effect on HCC, which was associated with the infiltration of CD8^+^T cells by BJJP via CCL5.

BJJP is used to treat hepatic cirrhosis and HCC in most Chinese hospitals utilizing TCM. Monotherapy with BJJP or combination with modern medicine has benefited numerous patients in Asia. However, the lack of clinical studies limits the understanding of the cellular and molecular mechanisms underlying the effects of BJJP on HCC. The present study demonstrated that BJJP facilitates the expression of CCL5, resulting in the infiltration of CD8^+^ T cells into tumors. Moreover, whether BJJP had a similar effect on human HCC cells was also assessed. The BJJP-treated cells showed increased levels of CCL5 expression, consistent with the results obtained in mice studies. This suggests that BJJP may have similar effects clinically.

In summary, the findings from this study provide new insights into the anticancer effects of BJJP, which exerted its effects by regulating the expression of CCL5. These results, therefore, suggest that BJJP has great potential application in the clinical treatment of HCC.

## Data Availability

The original contributions presented in the study are included in the article/Supplementary Material, further inquiries can be directed to the corresponding authors.

## References

[B1] Aghajani M. J. Yang T. Schmitz U. James A. Mccafferty C. E. de Souza P. (2020). Epithelial-to-mesenchymal Transition and its Association with PD-L1 and CD8 in Thyroid Cancer. Endocr. Connect. 9 (10), 1028–1041. 10.1530/EC-20-0268 33112841 PMC7707834

[B2] An H. Lin J. Sun H. Xu L. Su J. He C. (2018). Biejiajian Pills Inhibits Hepatoma Carcinoma Cell Vasculogenic Mimicry by Suppressing RhoA/ROCK Signaling Pathway. Nan Fang Yi Ke Da Xue Xue Bao 38 (8), 997–1001. 10.3969/j.issn.1673-4254.2018.08.16 30187871 PMC6744031

[B3] Berghuis D. Santos S. J. Baelde H. J. Taminiau A. H. Egeler R. M. Schilham M. W. (2011). Pro-inflammatory Chemokine-Chemokine Receptor Interactions within the Ewing Sarcoma Microenvironment Determine CD8(+) T-Lymphocyte Infiltration and Affect Tumour Progression. J. Pathol. 223 (3), 347–357. 10.1002/path.2819 21171080

[B4] Carstens J. L. Correa de Sampaio P. Yang D. Barua S. Wang H. Rao A. (2017). Spatial Computation of Intratumoral T Cells Correlates with Survival of Patients with Pancreatic Cancer. Nat. Commun. 8, 15095. 10.1038/ncomms15095 28447602 PMC5414182

[B5] Chae Y. K. Chang S. Ko T. Anker J. Agte S. Iams W. (2018). Epithelial-mesenchymal Transition (EMT) Signature Is Inversely Associated with T-Cell Infiltration in Non-small Cell Lung Cancer (NSCLC). Sci. Rep. 8 (1), 2918. 10.1038/s41598-018-21061-1 29440769 PMC5811447

[B6] Dangaj D. Bruand M. Grimm A. J. Ronet C. Barras D. Duttagupta P. A. (2019). Cooperation between Constitutive and Inducible Chemokines Enables T Cell Engraftment and Immune Attack in Solid Tumors. Cancer Cell. 35 (6), 885. 10.1016/j.ccell.2019.05.004 31185212 PMC6961655

[B7] Fan L. Qiu D. Huang G. Chen J. Wu Q. Xiong S. (2020). Wogonin Suppresses IL-10 Production in B Cells via STAT3 and ERK Signaling Pathway. J. Immunol. Res. 2020, 3032425. 10.1155/2020/3032425 32566686 PMC7285295

[B8] Fu J. Xu D. Liu Z. Shi M. Zhao P. Fu B. (2007). Increased Regulatory T Cells Correlate with CD8 T-Cell Impairment and Poor Survival in Hepatocellular Carcinoma Patients. Gastroenterology 132 (7), 2328–2339. 10.1053/j.gastro.2007.03.102 17570208

[B9] Galon J. Costes A. Sanchez-Cabo F. Kirilovsky A. Mlecnik B. Lagorce-Pagès C. (2006). Type, Density, and Location of Immune Cells within Human Colorectal Tumors Predict Clinical Outcome. Science 313 (5795), 1960–1964. 10.1126/science.1129139 17008531

[B10] Gao Y. Guo W. Geng X. Zhang Y. Zhang G. Qiu B. (2020). Prognostic Value of Tumor-Infiltrating Lymphocytes in Esophageal Cancer: an Updated Meta-Analysis of 30 Studies with 5,122 Patients. Ann. Transl Med. 8 (13), 822. 10.21037/atm-20-151 32793667 PMC7396260

[B11] Gou H. Huang R.-C. Zhang F.-L. Su Y.-H. (2021). Design of Dual Targeting Immunomicelles Loaded with Bufalin and Study of Their Anti-tumor Effect on Liver Cancer. J. Integr. Med. 19 (5), 408–417. 10.1016/j.joim.2021.05.001 34130942

[B12] Haque M. A. Jantan I. Arshad L. Bukhari S. N. A. (2017). Exploring the Immunomodulatory and Anticancer Properties of Zerumbone. Food Funct. 8 (10), 3410–3431. 10.1039/c7fo00595d 28714500

[B13] Jang S. M. Yee S. T. Choi J. Choi M. S. Do G. M. Jeon S. M. (2009). Ursolic Acid Enhances the Cellular Immune System and Pancreatic Beta-Cell Function in Streptozotocin-Induced Diabetic Mice Fed a High-Fat Diet. Int. Immunopharmacol. 9 (1), 113–119. 10.1016/j.intimp.2008.10.013 19013541

[B14] Ju S. A. Park S. M. Lee Y. S. Bae J. H. Yu R. An W. G. (2012). Administration of 6-gingerol Greatly Enhances the Number of Tumor-Infiltrating Lymphocytes in Murine Tumors. Int. J. Cancer. 130 (11), 2618–2628. 10.1002/ijc.26316 21792901

[B15] Kim J. S. Jeong S. K. Oh S. J. Lee C. G. Kang Y. R. Jo W. S. (2020). The Resveratrol Analogue, HS-1793, E-nhances the E-ffects of R-adiation T-herapy through the I-nduction of A-nti-tumor I-mmunity in M-ammary T-umor G-rowth. Int. J. Oncol. 56 (6), 1405–1416. 10.3892/ijo.2020.5017 32236622 PMC7170036

[B16] Kondratiev S. Sabo E. Yakirevich E. Lavie O. Resnick M. B. (2004). Intratumoral CD8+ T Lymphocytes as a Prognostic Factor of Survival in Endometrial Carcinoma. Clin. Cancer Res. 10 (13), 4450–4456. 10.1158/1078-0432.CCR-0732-3 15240536

[B17] Kumari R. Sahu M. K. Tripathy A. Uthansingh K. Behera M. (2018). Hepatocellular Carcinoma Treatment: Hurdles, Advances and Prospects. Hepat. Oncol. 5 (2), HEP08. 10.2217/hep-2018-0002 31293776 PMC6613045

[B18] Li J.-J. Liang Q. Sun G.-C. (2021). Traditional Chinese Medicine for Prevention and Treatment of Hepatocellular Carcinoma: A Focus on Epithelial-Mesenchymal Transition. J. Integr. Med. 19, 469–477. 10.1016/j.joim.2021.08.004 34538644

[B19] Liao X. Bu Y. Jia Q. (2020). Traditional Chinese Medicine as Supportive Care for the Management of Liver Cancer: Past, Present, and Future. Genes Dis. 7 (3), 370–379. 10.1016/j.gendis.2019.10.016 32884991 PMC7452431

[B20] Ling A. Edin S. Wikberg M. L. Öberg Å. Palmqvist R. (2014). The Intratumoural Subsite and Relation of CD8(+) and FOXP3(+) T Lymphocytes in Colorectal Cancer Provide Important Prognostic Clues. Br. J. Cancer 110 (10), 2551–2559. 10.1038/bjc.2014.161 24675384 PMC4021513

[B21] Ling C. Q. Fan J. Lin H. S. Shen F. Xu Z. Y. Lin L. Z. (2018). Clinical Practice Guidelines for the Treatment of Primary Liver Cancer with Integrative Traditional Chinese and Western Medicine. J. Integr. Med. 16 (4), 236–248. 10.1016/j.joim.2018.05.002 29891180

[B22] Llovet J. M. Kelley R. K. Villanueva A. Singal A. G. Pikarsky E. Roayaie S. (2021). Hepatocellular Carcinoma. Nat. Rev. Dis. Primers 7 (1), 6. 10.1038/s41572-020-00240-3 33479224 PMC13537433

[B23] Mahmoud S. M. Paish E. C. Powe D. G. Macmillan R. D. Grainge M. J. Lee A. H. (2011). Tumor-infiltrating CD8+ Lymphocytes Predict Clinical Outcome in Breast Cancer. J. Clin. Oncol. 29 (15), 1949–1955. 10.1200/JCO.2010.30.5037 21483002

[B24] Mohs A. Kuttkat N. Reissing J. Zimmermann H. W. Sonntag R. Proudfoot A. (2017). Functional Role of CCL5/RANTES for HCC Progression during Chronic Liver Disease. J. Hepatol. 66 (4), 743–753. 10.1016/j.jhep.2016.12.011 28011329

[B25] Ping L. I. Zhou Y. M. Xie T. X. Fan L. Cao J. X. (2008). Clinical Study of Biejiajian Decoction on Fibroid Degeneration of Liver Induced by Interventional Radiological Therapy for Liver Cancer. J. Traditional Chin. Med. Univ. Hunan 1 (28), 2. 10.1128/JVI.77.24.13348-13360.2003

[B26] Qi F. Zhao L. Zhou A. Zhang B. Li A. Wang Z. (2015). The Advantages of Using Traditional Chinese Medicine as an Adjunctive Therapy in the Whole Course of Cancer Treatment Instead of Only Terminal Stage of Cancer. Biosci. Trends. 9 (1), 16–34. 10.5582/bst.2015.01019 25787906

[B27] Sharma P. Shen Y. Wen S. Yamada S. Jungbluth A. A. Gnjatic S. (2007). CD8 Tumor-Infiltrating Lymphocytes Are Predictive of Survival in Muscle-Invasive Urothelial Carcinoma. Proc. Natl. Acad. Sci. U S A. 104 (10), 3967–3972. 10.1073/pnas.0611618104 17360461 PMC1820692

[B28] Singh S. K. Mishra M. K. Eltoum I. A. Bae S. Lillard J. W. Singh R. (2018). CCR5/CCL5 axis Interaction Promotes Migratory and Invasiveness of Pancreatic Cancer Cells. Sci. Rep. 8 (1), 1323. 10.1038/s41598-018-19643-0 29358632 PMC5778036

[B29] Sun H. He S. Wen B. Jia W. Fan E. Zheng Y. (2014). Effect of Biejiajian Pills on Wnt Signal Pathway Molecules β-catenin and GSK-3β and the Target Genes CD44v6 and VEGF in Hepatocellular Carcinoma Cells. Nan Fang Yi Ke Da Xue Xue Bao 34 (10), 1454–1458. 10.3969/j.issn.1673-4254.2014.10.11 25345941

[B30] Sun J. Chen W. Wen B. Zhang M. Sun H. Yang X. (2021). Biejiajian Pill Inhibits Carcinogenesis and Metastasis via the Akt/GSK-3β/Snail Signaling Pathway in Hepatocellular Carcinoma. Front. Pharmacol. 12, 610158. 10.3389/fphar.2021.610158 33762939 PMC7982731

[B31] Tang K. Y. Du S. L. Wang Q. L. Zhang Y. F. Song H. Y. (2020). Traditional Chinese Medicine Targeting Cancer Stem Cells as an Alternative Treatment for Hepatocellular Carcinoma. J. Integr. Med. 18 (3), 196–202. 10.1016/j.joim.2020.02.002 32067923

[B32] Xu L. M. Liu P. (2020). Guidelines for Diagnosis and Treatment of Hepatic Fibrosis with Integrated Traditional Chinese and Western Medicine (2019 Edition). J. Integr. Med. 18 (3), 203–213. 10.1016/j.joim.2020.03.001 32331978

[B33] Xue D. Zheng Y. Wen J. Han J. Tuo H. Liu Y. (2021). Role of Chemokines in Hepatocellular Carcinoma (Review). Oncol. Rep. 45 (3), 809–823. 10.3892/or.2020.7906 33650640 PMC7859922

[B34] Yang W. Bai Y. Xiong Y. Zhang J. Chen S. Zheng X. (2016). Potentiating the Antitumour Response of CD8(+) T Cells by Modulating Cholesterol Metabolism. Nature 531 (7596), 651–655. 10.1038/nature17412 26982734 PMC4851431

[B35] Yang Z. Liao X. Lu Y. Xu Q. Tang B. Chen X. (2017). Add-On Therapy with Traditional Chinese Medicine Improves Outcomes and Reduces Adverse Events in Hepatocellular Carcinoma: A Meta-Analysis of Randomized Controlled Trials. Evid. Based Complement. Alternat Med. 2017, 3428253. 10.1155/2017/3428253 28680448 PMC5478821

[B36] Yao S. Y. (2009). Modified Biejia Decoction Theraphtic Effect on 54 Patients with Primary Liver Cancer. J. Liaoning Univ. Traditional Chin. Med. 6 (11), 2.

[B37] Zhang S. Zhong M. Wang C. Xu Y. Gao W. Q. Zhang Y. (2018). CCL5-deficiency Enhances Intratumoral Infiltration of CD8+ T Cells in Colorectal Cancer. Cell Death Dis. 9 (7), 766. 10.1038/s41419-018-0796-2 29991744 PMC6039518

[B38] Zhang Y. Guan X. Y. Jiang P. (2020). Cytokine and Chemokine Signals of T-Cell Exclusion in Tumors. Front. Immunol. 11, 594609. 10.3389/fimmu.2020.594609 33381115 PMC7768018

[B39] Zhao H. T. Meng Y. B. Zhai X. F. Cheng B. B. Yu S. S. Yao M. (2020). Comparable Effects of Jiedu Granule, a Compound Chinese Herbal Medicine, and Sorafenib for Advanced Hepatocellular Carcinoma: A Prospective Multicenter Cohort Study. J. Integr. Med. 18 (4), 319–325. 10.1016/j.joim.2020.05.003 32532615

[B40] Zheng K. Mengguo H. E. Wang Z. Tan D. (2017). Effect of Biejiajian Pills and Aidi Injection Combined Transcatheter Arterial Chemoembolization for Moderate or Advanced Liver Cancer. Anhui Med. Pharm. J. 21 (10), 1909–1912. 10.3969/j.issn.1009-6469.2017.10.043

