# Supplementary material for: Biejiajian Pill Promotes the Infiltration of CD8+ T Cells in Hepatocellular Carcinoma by Regulating the Expression of CCL5
Source: Front Pharmacol. 2021 Nov 26;12:771046. doi: 10.3389/fphar.2021.771046 (PMC8661106; doi:10.3389/fphar.2021.771046)
Supplement: Supplementary file 1 [file DataSheet4.docx]

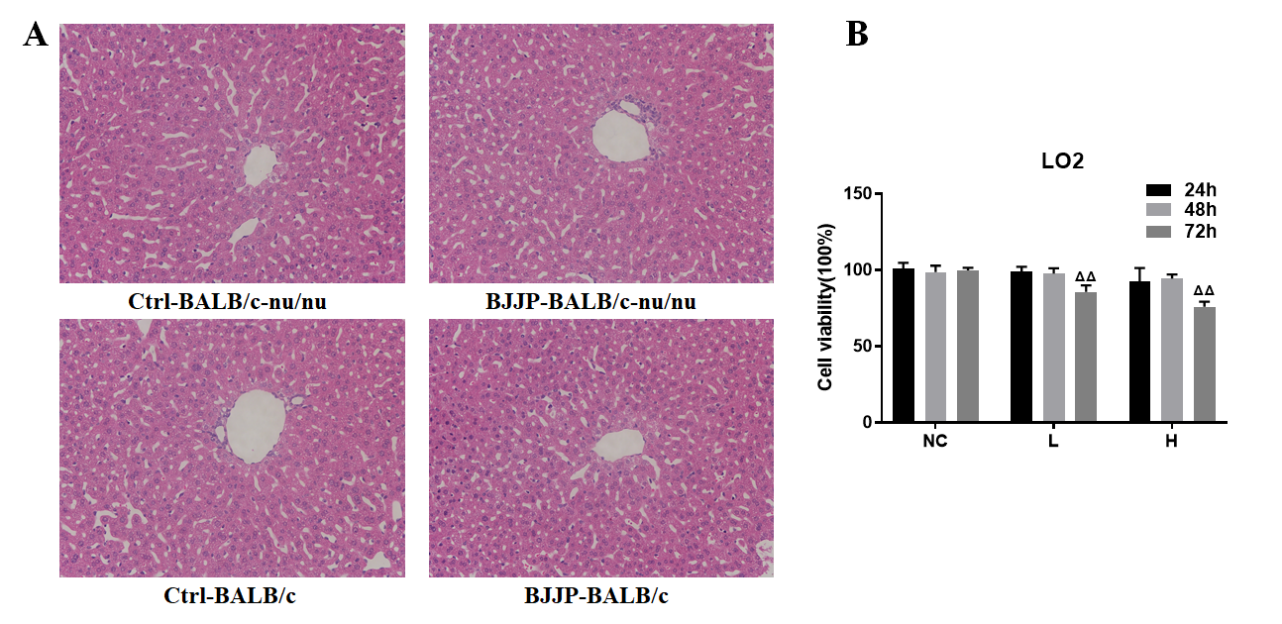


**Supplemental Figure 4. BJJP shows less cytotoxicity on liver tissues and** **immortalized liver LO_2_ cells.**

(A) HE staining of liver tissues after BJJP treatment. (B) CCK8 analysis was used to detect the effect of BJJP on immortalized liver LO_2_ cells. Data is presented in terms of mean ± SD (n=3). **^Δ^***P* < 0.05 vs controls.
