# Supplementary material for: Biejiajian Pill Promotes the Infiltration of CD8+ T Cells in Hepatocellular Carcinoma by Regulating the Expression of CCL5
Source: Front Pharmacol. 2021 Nov 26;12:771046. doi: 10.3389/fphar.2021.771046 (PMC8661106; doi:10.3389/fphar.2021.771046)
Supplement: Supplementary file 2 [file Table1.docx]

Supplementary Material

# Mouse and human specific gene primers

| **Primer name** | **Sequence (5'to3')** |
| --- | --- |
| **mmu-** CCL5**-F** | TGCCCACGTCAAGGAGTATTTC |
| **mmu-** CCL5**-R** | AACCCACTTCTTCTCTGGGTTG |
| **mmu-** CCL9**-F** | CCCTCTCCTTCCTCATTCTTACA |
| **mmu-** CCL9**-R** | AGTCTTGAAAGCCCATGTGAAA |
| **mmu-** CCL17**-F** | CGAGAGTGCTGCCTGGATTACT |
| **mmu-** CCL17**-R** | GGTCTGCACAGATGAGCTTGCC |
| **mmu-** CXCL9**-F** | CCTAGTGATAAGGAATGCACGATG |
| **mmu-** CXCL9**--R** | CTAGGCAGGTTTGATCTCCGTTC |
| **mmu-** CXCL10**-F** | ATCATCCCTGCGAGCCTATCCT |
| **mmu-** CXCL10**-R** | GACCTTTTTTGGCTAAACGCTTTC |
| **mmu-** CXCL12**-F** | CATCCATCCATCCATCCA |
| **mmu-** CXCL12**-R** | TTCAGGGTCATGGAGACAGT |
| **hsa-CCL5-F** | CCTGCTGCTTTGCCTACATTGC |
| **hsa-CCL5-R** | ACACACTTGGCGGTTCTTTCGG |
