# Supplementary material for: Biejiajian Pill Promotes the Infiltration of CD8+ T Cells in Hepatocellular Carcinoma by Regulating the Expression of CCL5
Source: Front Pharmacol. 2021 Nov 26;12:771046. doi: 10.3389/fphar.2021.771046 (PMC8661106; doi:10.3389/fphar.2021.771046)
Supplement: Supplementary file 3 [file DataSheet3.docx]

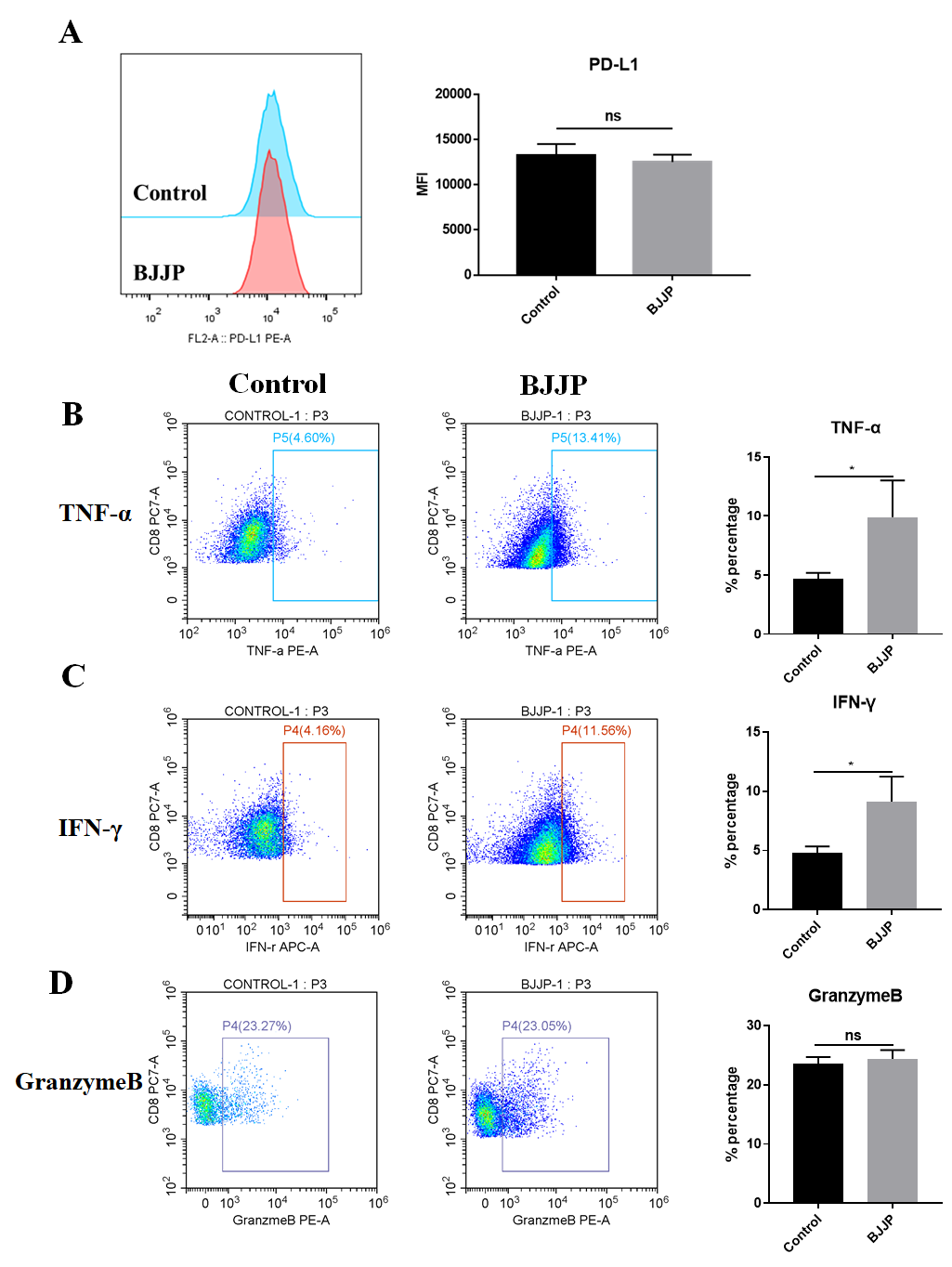


**Supplemental Figure 3. BJJP promotes the effector function of CD8^+^T cells *In Vivo*.** (A) Intracellular PD-L1 staining on tumor cells was performed. (B-D) Intracellular cytokine TNF-α, IFN-γ, GranzymeB expression on CD8^+^ T cells were detected with Flow cytometry. Data is presented in terms of mean ± SD (n=3). ******P* < 0.05 vs controls.
