# Supplementary material for: Biejiajian Pill Promotes the Infiltration of CD8+ T Cells in Hepatocellular Carcinoma by Regulating the Expression of CCL5
Source: Front Pharmacol. 2021 Nov 26;12:771046. doi: 10.3389/fphar.2021.771046 (PMC8661106; doi:10.3389/fphar.2021.771046)
Supplement: Supplementary file 4 [file DataSheet2.docx]

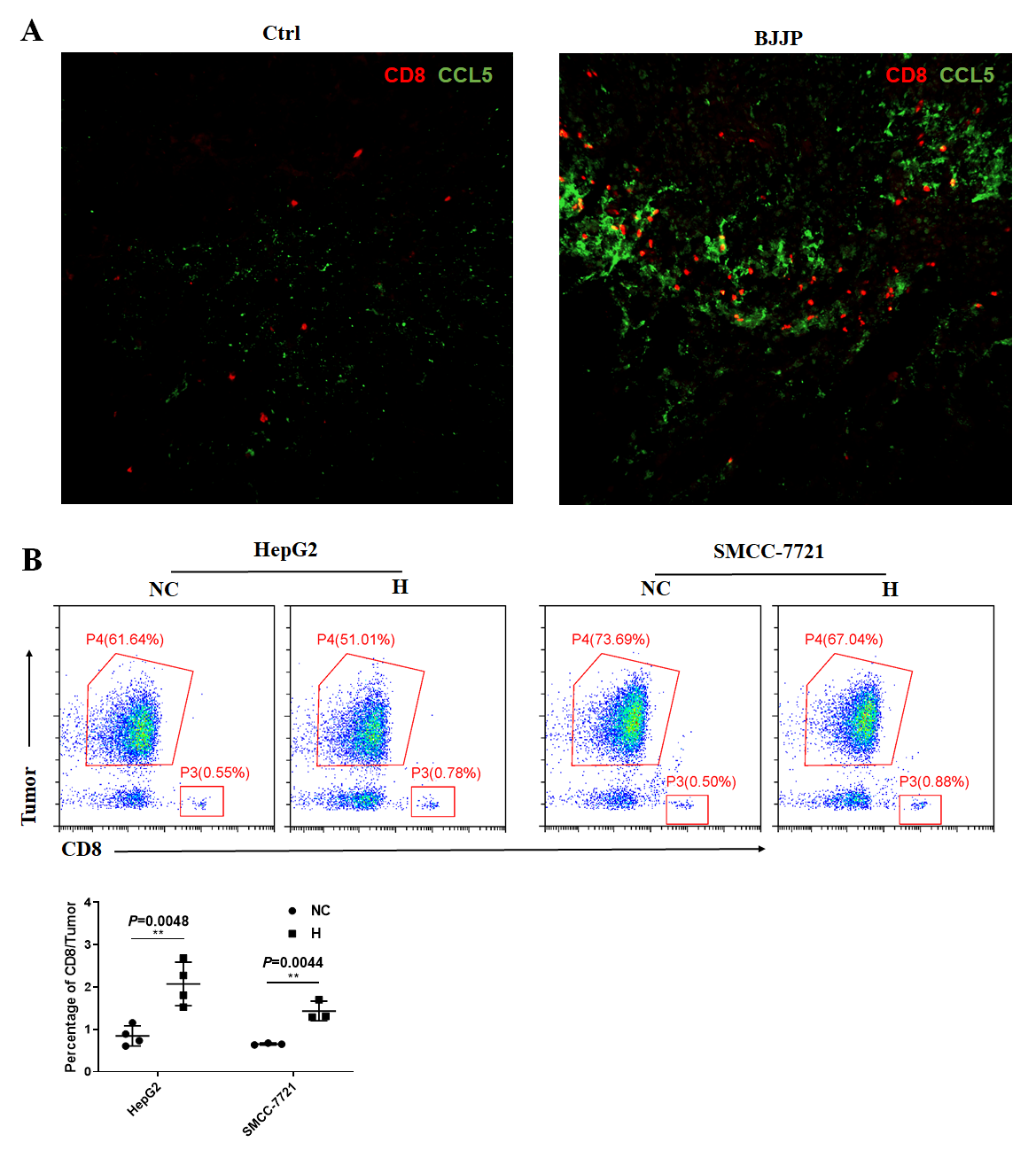


**Supplemental Figure 2. BJJP promotes the infiltration of CD8^+^ T cells *In Vitro*.** (A)immunostaining of CD8^+^T cells and CCL5 between control and BJJP treated group using subcutaneous tumor samples. (B) The migrating human PBMC CD8^+^ T cell were detected with Flow cytometry. Data is presented in terms of mean ± SD (n=3). **P* < 0.05, ***P* < 0.01 vs controls.
