# Supplementary material for: Biejiajian Pill Promotes the Infiltration of CD8+ T Cells in Hepatocellular Carcinoma by Regulating the Expression of CCL5
Source: Front Pharmacol. 2021 Nov 26;12:771046. doi: 10.3389/fphar.2021.771046 (PMC8661106; doi:10.3389/fphar.2021.771046)
Supplement: Supplementary file 5 [file DataSheet1.docx]

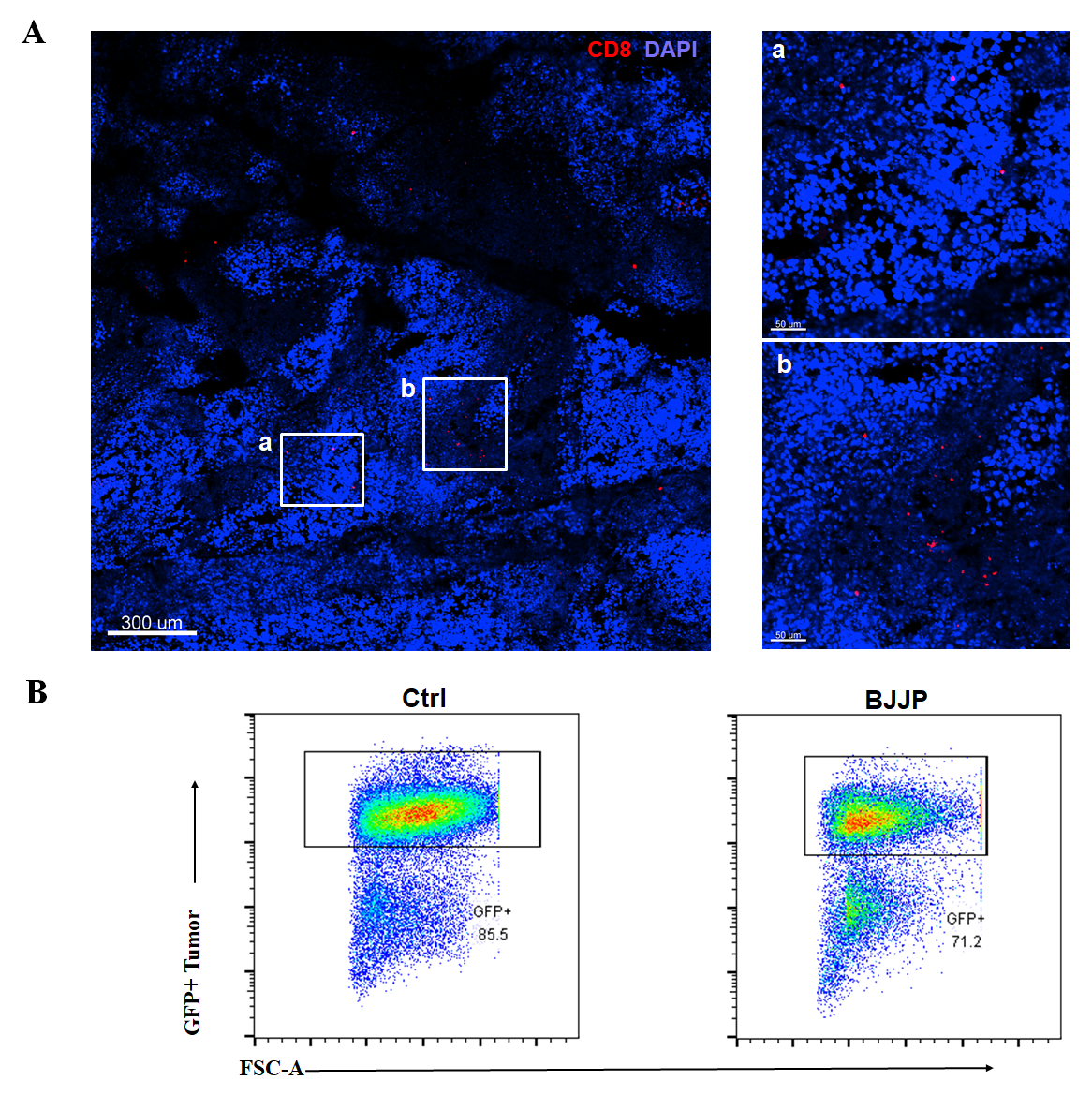


**Supplemental Figure 1. BJJP promotes the infiltration of CD8+ T cells in vivo.** (A)immunostaining of CD8^+^T cells in control group. (B) Gating dot plot to indicate the purity of GFP^+^ cell from control and BJJP treated group for cell sorting.
